# Supplementary material for: Investigation of Novel Therapeutic Targets for Rheumatoid Arthritis Through Human Plasma Proteome
Source: Biomedicines. 2025 Jul 29;13(8):1841. doi: 10.3390/biomedicines13081841 (PMC12383807; doi:10.3390/biomedicines13081841)
Supplement: Supplementary file 1 [file biomedicines-13-01841-s001.zip › Supplementary File S2.pdf]

Investigation of Novel Therapeutic Targets for Rheumatoid Arthritis Through Human Plasma Proteome

Supplementary File S2

Data integration of plasma proteins

The protein names, uniport IDs and gene symbols were cross-mapped on annotations from each study, with manual verification. All single nucleotide polymorphisms (SNPs) and genes were aligned to the human genome Build 37 (NCBI GRCh37) to standardize genomic coordinates. The curation and integration of protein quantitative trait loci (pQTLs) followed these steps: (1) Extract SNPs that were genetically associated with corresponding proteins at a genome-wide significance level ($p < 5.000\times10^{-8}$); (2) Exclude SNPs located within the Major Histocompatibility Complex (MHC) region (chr6: 26.0–34.0 Mb) due to complex linkage disequilibrium (LD) structure; (3) Perform LD clumping ($r^2 < 0.001$, ±10,000 kb) to identify independent pQTLs for each protein; (4) Classify pQTLs into cis-pQTLs (located within 1000 kb of transcription start site of the protein-coding gene) and trans-pQTLs (located outside this window); (5) Calculate R^2 ($R^2 = 2 \times EAF \times (1 - EAF) \times \beta^2$) and F statistics ($F = R^2 \times (N - 2)/(1 - R^2)$) for each SNP to assess proportion of variance explained (PVE) and statistical strength. For proteins represented across multiple studies, SNPs from the study with the largest sum of R^2 were selected.

Proteome-wide mendelian randomization (Pro-MR) analysis

In MR analyses across this study, Wald ratio method (for single instrumental variable [IV]) and inverse variance weighted (IVW) method (for multiple IVs) were employed as primary methods to estimate causal effects. The effect size (ES) was interpreted as change in the outcome (measured by β for continuous outcomes or by natural log of odds ratio (lnOR) for binary outcomes) per one standard deviation (SD) increase in the exposure variable. Two datasets containing cis-pQTLs and all pQTLs were analyzed separately. In discovery MR,

multiple sensitivity analyses were performed for proteins with sufficient IVs to enhance robustness and reliability of the primary findings. Steiger filtering method was used as a directional test to identify whether reverse causality had distorted the results. A "TRUE" result indicated a correct causal direction from the exposure to the outcome. Furthermore, Heterogeneity and horizontal pleiotropy across multiple IVs were assessed using Cochrane's Q statistic and MR-Egger method, with $P < 0.050$ indicating the presence of heterogeneity or horizontal pleiotropy. In Replication MR, pQTLs not available in the replication dataset were proxied by SNPs with high LD ($r^2 > 0.800$). In Reverse MR, IVs proxying rheumatoid arthritis (RA) were selected according to the same criteria applied for pQTLs.

Colocalization analysis

Colocalization was conducted by considering a 500 kb genetic region on either side of each pQTL, with prior probabilities set as $P_1 = 1.000 \times 10^{-4}$ (the probability that a SNP is associated with the protein), $P_2 = 1.000 \times 10^{-4}$ (the probability that a SNP is associated with RA), and $P_{12} = 1.000 \times 10^{-5}$ (the probability that a SNP is associated with both protein and RA). Approximate Bayes factor colocalization (coloc.abf) served as the primary method. However, to address its limitation in distinguishing multiple shared causal hits between exposure and outcome, we supplemented with sum of single effects colocalization (coloc.susie). The analysis generated posterior probabilities (PPs) for five hypotheses: H_0: neither trait has a genetic association; H_1: only the exposure has a genetic association; H_2: only the outcome has a genetic association; H_3: both traits are associated but with distinct causal variants; and H_4: both traits are associated and share a single causal variant. The stronger evidence from either method was prioritized. Once a protein was proxied by more than one pQTL, the final result was determined by the pQTL showing the strongest evidence.

Transcriptome-wide summary-data-based MR (SMR)

To assess potential heterogeneity of gene effects in local or systemic compartments, we included extensive expression quantitative trait locus (eQTL) datasets covering various tissues

from eQTLGen Consortium, Consortium for the Architecture of Gene Expression (CAGE), Westra et al., PsychENCODE consortium, and Genotype-Tissue Expression (GTEx) project version 8 release. All parameters were kept at default. When multiple target SNPs were associated with the same probe, we prioritized the result with the lowest *p*-value.

Pleiotropy assessment

The pleiotropy was assessed as follows: Step 1: Identify whether each trans-pQTL is significantly associated ($p < 5.000 \times 10^{-8}$) with any other gene (secondary gene) at protein or gene expression level, using data from the corresponding study or eQTLGen. Step 2: Establish IVs for secondary genes using associated ($p < 5.000 \times 10^{-8}$) and independent cis-pQTLs (at protein level) from the same study. For secondary genes identified via eQTLGen, cis-pQTLs were sourced from data published by Ferkingstad et al., which offers the most extensive protein data. Where cis-pQTLs were unavailable, we used the lead cis-eQTL (at gene expression level) identified in eQTLGen as a substitute. Step 3: Conduct MR analyses to examine the association between secondary genes and RA using the discovery RA dataset. The significance threshold was adjusted to $p < 2.404 \times 10^{-4}$ (0.050/208). Step 4: Map risk secondary genes identified in Step 3 to Reactome platform (https://reactome.org/) and Kyoto Encyclopedia of Genes and Genomes (KEGG) pathways to assess functional overlap with the corresponding prioritized proteins. Functional overlap suggests potential vertical pleiotropy, whereas its absence indicates likely horizontal pleiotropy.

Cell-type-specific expression analysis

The single-cell RNA sequencing (scRNA-seq) data was generated from Zhang et al. [1], based on 36 RA synovial samples and 15 osteoarthritis (OA) synovial samples. All tissue samples had synovial lining documented by histology. We performed quality control and preprocessing on the raw data. Genes with nonzero expression in fewer than 10 cells and cells with fewer than 1000 detected genes were excluded. Additionally, cells with over 25% of molecules derived from mitochondrial genes were discarded. The data were then normalized, scaled,

dimensionally reduced, clustered, and annotated by cell type using definitions from the original study. The synovial cell composition and gene expression profiles were then analyzed. Genes with a log2 fold change (FC) > 0.500 and a false discovery rate adjusted P value (P_{FDR}) < 0.050 were identified as enriched in specific cell types using Wilcoxon Rank Sum test. Further, we explored intergroup differences in the expression of these cell-type-enriched genes between RA and OA using Wilcoxon Rank Sum test.

Mediation analysis

First, univariate MR was applied to systematically identify modifiable factors significantly influencing both RA (Total effect) and prioritized proteins (Effect A). IVs for modifiable factors were selected in the same manner as pQTLs. Due to the lack of available IVs for most gut microbiome phenotypes at $p < 5.000\times10^{-8}$, we used a more lenient threshold of $p < 5.000 \times 10^{-6}$ [2]. Subsequently, multivariate MR (MVMR) was used to assess the independent effects of proteins on RA (Effect B) after adjustment for the corresponding modifiable factors. IV selection in MVMR followed the same criteria as univariate MR. For modifiable factor-protein-RA pairs showing potential mediating associations, mediation effects were calculated as: Mediation effect = Effect A × Effect B. Standard errors (SE) were estimated using delta method, and the P value for mediation effects was derived. Proportion mediated was calculated as: Proportion mediated = (Mediation effect / Total effect) × 100%. For above analyses, the significance thresholds were all set at $p < 0.050$ to maximize findings.

References

[1] Zhang F, Wei K, Slowikowski K, Fonseka CY, Rao DA, Kelly S, et al. Defining inflammatory cell states in rheumatoid arthritis joint synovial tissues by integrating single-cell transcriptomics and mass cytometry. *Nat. Immunol.* 2019;20:928–42. https://doi.org/10.1038/s41590-019-0378-1.

[2] Sun J, Zhao J, Zhou S, Li X, Li T, Wang L, et al. Systematic investigation of genetically determined plasma and urinary metabolites to discover potential interventional targets for colorectal cancer. *J. Natl. Cancer Inst.* 2024;116:1303–12. https://doi.org/10.1093/jnci/djae089.
