# Supplementary material for: Investigation of Novel Therapeutic Targets for Rheumatoid Arthritis Through Human Plasma Proteome
Source: Biomedicines. 2025 Jul 29;13(8):1841. doi: 10.3390/biomedicines13081841 (PMC12383807; doi:10.3390/biomedicines13081841)

Investigation of novel therapeutic targets for rheumatoid arthritis through human plasma proteome

Supplementary File S3

Figure S1. Feature plots showing expression profiles of prioritized protein-coding genes in specific synovial cell types.

Figure S2. Intergroup comparisons reveal no significant differences in expression levels of these prioritized protein-coding genes in their respective enriched cell types (noted in parentheses) between RA and OA groups.

Figure S3. Mediating role of several prioritized proteins in the associations between modifiable factors and RA.

Figure S1. Feature plots showing expression profiles of prioritized protein-coding genes in specific synovial cell types.

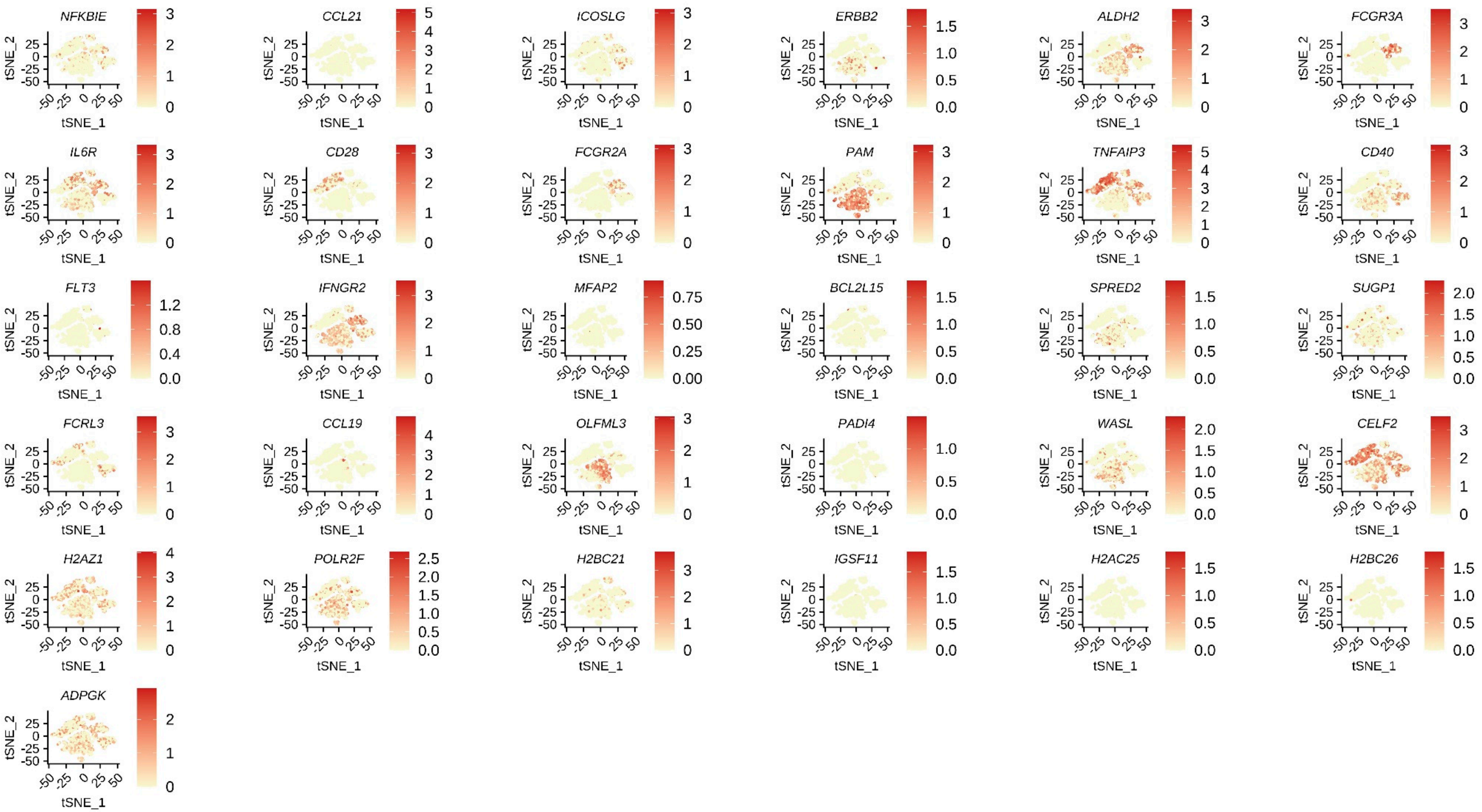

Figure S2. Intergroup comparisons reveal no significant differences in expression levels of these prioritized protein-coding genes in their respective enriched cell types (noted in parentheses) between RA and OA groups. Abbreviations: ns, not significant; RA, rheumatoid arthritis; OA, osteoarthritis.

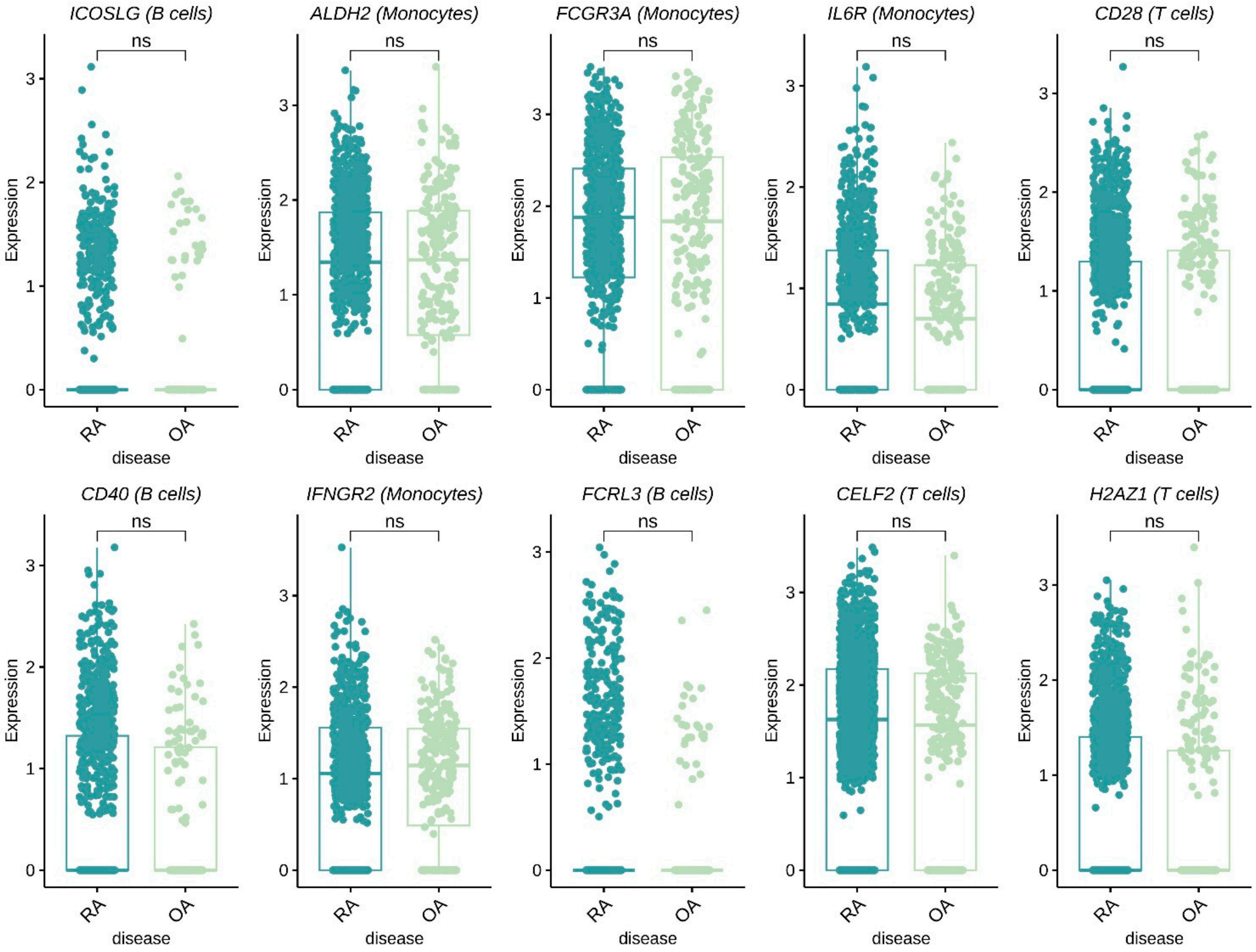

Figure S3. Mediating role of several prioritized proteins in the associations between modifiable factors and RA. (**A**) SUGP1 as a partial mediator of the effect of Eubacterium brachy on RA. (**B**) IGSF11 as a partial mediator of the effect of Holdemania on RA. (**C**) H2AC25 as a partial mediator of the effect of Holdemania on RA. (**D**) POLR2F as a partial mediator of the effect of BMI on RA. (**E**) ALDH2 as a partial mediator of the effect of BMI on RA. (**F**) FCRL3 as a partial mediator of the effect of BMI on RA. Abbreviations: ES, effect size; RA, rheumatoid arthritis; BMI, body mass index.

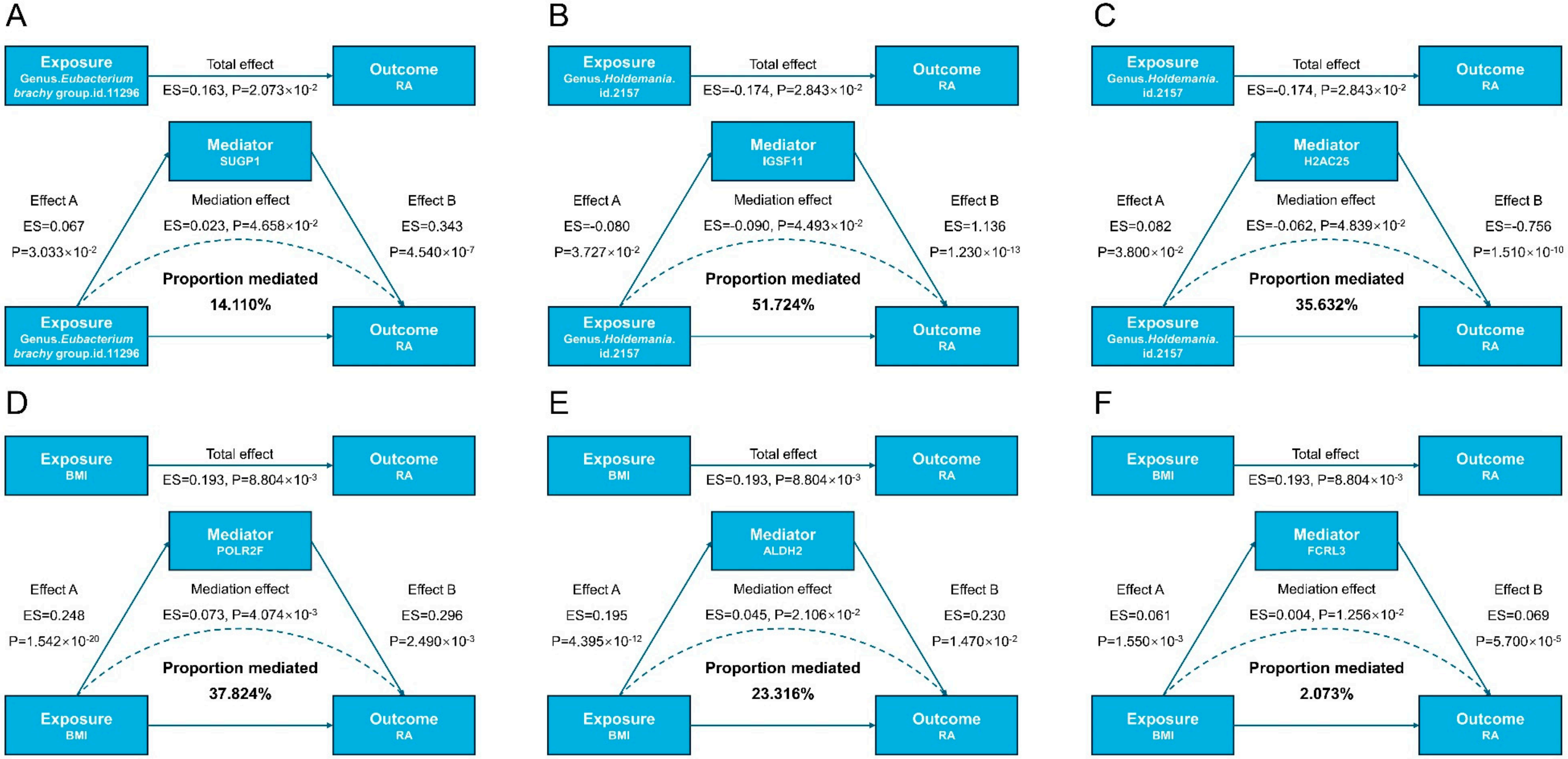

Supplement: Supplementary file 1 [file biomedicines-13-01841-s001.zip › Supplementary File S3.pdf]
